# Long-term cigarette smoke exposure inhibits histone deacetylase 2 expression and enhances the nuclear factor-κB activation in skeletal muscle of mice

**DOI:** 10.18632/oncotarget.18089

**Published:** 2017-05-23

**Authors:** Dongmei Huang, Zhiying Ma, Yili He, Ying Xiao, Honglin Luo, Qiuli Liang, Xiaoning Zhong, Jing Bai, Zhiyi He

**Affiliations:** ^1^ Department of Respiratory Medicine, The First Affiliated Hospital of Guangxi Medical University, Nanning, Guangxi, 530021, China

**Keywords:** cigarette smoke exposure, muscle inflammation, histone deacetylase 2, nuclear factor-κB

## Abstract

Long-term cigarette smoke induces lung inflammatory injury and chronic obstructive pulmonary disease (COPD), associated with skeletal muscle inflammation. This study aimed at investigating how cigarette smoke promotes skeletal muscle inflammation and its molecular pathogenesis. Mice were exposed to air or cigarette smoke for 12 or 24 weeks, and C2C12 cells were stimulated with cigarette smoke extract (CSE). The mass and function, myotube formation, inflammatory cytokine production, histone deacetylase 2 (HDAC2) and nuclear factor-κB (NF-κB) p65 expression were detected in the gastrocnemius muscles of mice and C2C12 cells. In comparison with the control mice, cigarette smoke significantly damaged the lung and reduced the gastrocnemius muscle mass and body weights in mice. Cigarette smoke significantly down-regulated myosin heavy chain (MHC)-IIβ and HDAC2 expression, but enhanced NF-κBp65, keratinocyte chemoattractant (KC) and tumor necrosis factor (TNF)-α expression in the gastrocnemius muscles. CSE stimulation significantly inhibited the myotube formation, MyoD and HDAC2 expression, but enhanced NF-κBp65 expression, KC and TNF-α production in C2C12 cells, which were enhanced by HDAC2 knockdown and abrogated by a NF-κB inhibitor. CSE significantly inhibited the interaction of HDAC2 with NF-κBp65, and increased the levels of acetyl-NF-κBp65 in C2C12 cells. These data indicated that cigarette smoke inhibited HDAC2 expression and its interaction with NF-κBp65 to stimulate inflammation, contributing to the pathogenesis of COPD-related skeletal muscle atrophy in mice.

## INTRODUCTION

Long term heavy cigarette smoke (CS) is risk for the development of chronic obstructive pulmonary disease (COPD). Long-term CS induces inflammation, damages in the lung, result in extra-pulmonary comorbidities, such as skeletal muscle atrophy, coronary heart failure, depression, osteoporosis and obesity. Skeletal muscle atrophy and dysfunction is common in patients with COPD [1, 2]. It is associated with exacerbation of COPD and impairment of exercise tolerance, leading to a poor quality of life, and increased mortality rates in COPD patients [3, 4]. Hence, elucidating the molecular pathogenesis of COPD-related skeletal muscle atrophy will be of significance in development of new therapies.

A previous study has suggested that skeletal muscle atrophy is attributed to systemic inflammation mediated by the ‘‘spill-over’’ of inflammatory mediators into the circulation [5]. During the pathogenesis of COPD, inflammation predominantly damages the lungs. However, inflammatory mediators can ‘‘spill-over’’ into the circulation, which may result in systemic manifestations, such as skeletal muscle wasting. In patients with COPD, pro-inflammatory cytokines, like tumor necrosis factor (TNF)α, can activate the NF-κB signaling to increase inflammatory cytokine production and inducible nitric oxide synthase (iNOS) expression. These inflammatory mediators can facilitate the degradation of myosin heavy chain (MHC) through the ubiquitin-proteasome complex and muscle cell apoptosis. Therefore, during the progression of COPD, skeletal muscle atrophy is implicated by high levels of circulating TNF-α and other pro-inflammatory cytokines [6, 7]. Actually, activation of the NF-κB signaling can also increase production of TNF-α, keratinocyte chemoattractant (KC, IL-8) and others. Previous studies have shown that CS promotes NF-κBp65 expression, activates the NF-κB signaling and induces proinflammatory cytokine production by post-translational modifications of histone deacetylase in macrophages [8, 9]. CS or oxidants can inhibit activity and expression of histone deacetylase 2 (HDAC2) in the lung and enhance the process of histone acetylation. However, the mechanisms underlying the effect of long-term CS on the muscular morphology and mass, inflammation, the NF-κB activation, HDAC2 expression and activation have not been clarified *in vivo*.

In this study, we employed a mouse model of CS-related muscle atrophy and murine skeletal muscle C2C12 cells to test the effect of long-term CS on the mass, function, inflammation, the NF-κBp65 and HDAC2 expression in the gastrocnemius muscles of mice. Furthermore, we examined how CSE affected the myotube formation, inflammatory cytokine production, the NF-κBp65 and HDAC2 expression as well as the potential interaction between the NF-κBp65 and HDAC2 in differentiating C2C12 cells [10]. The results revealed that long-term CS induced the gastrocnemius muscle atrophy in mice, accompanied by inhibiting HDAC2 expression, but enhancing the NF-κB activation and proinflammatory cytokine production.

## RESULTS

### Long-term CS induces the lung injury and skeletal muscle atrophy in mice

**Figure 1:**
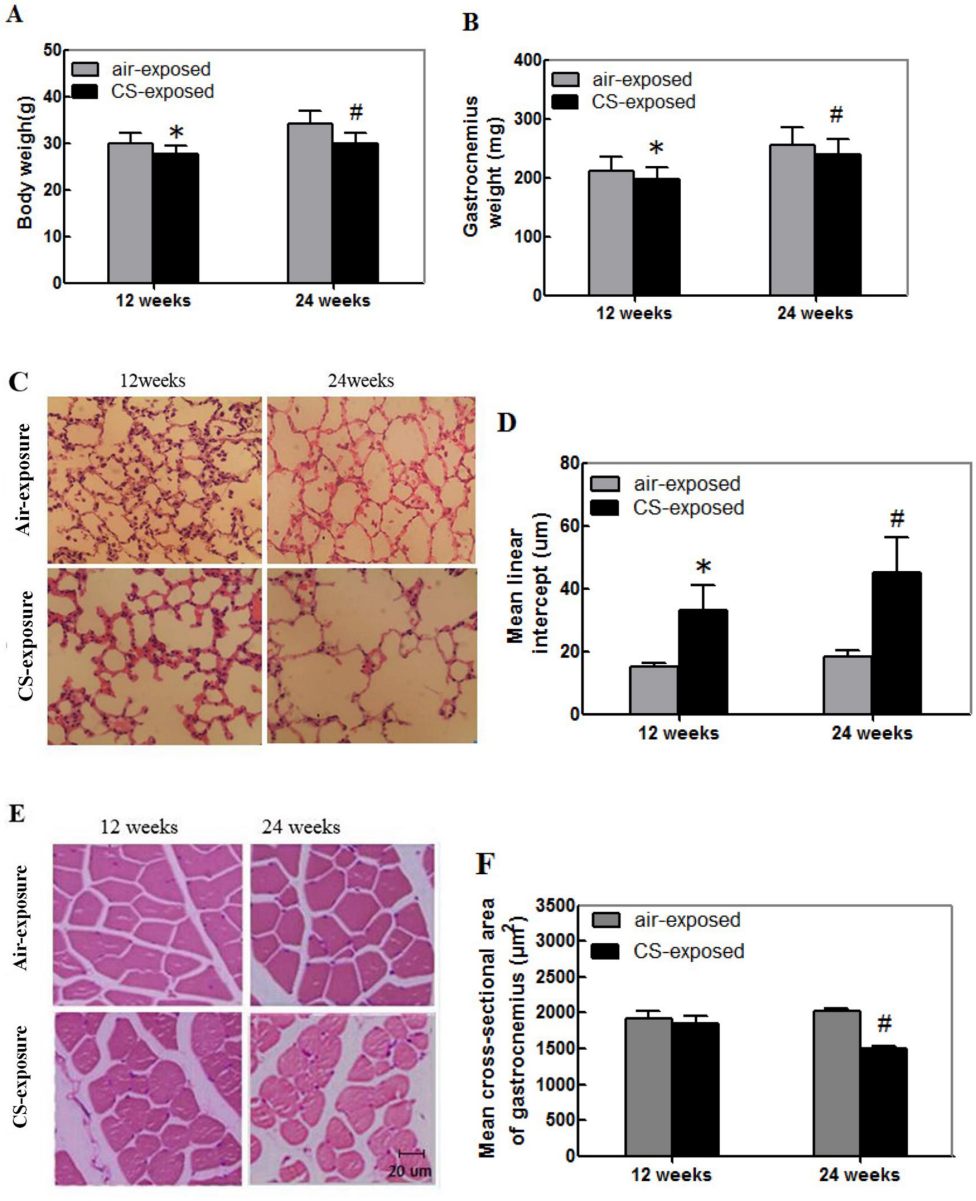
Cigarette smoking induces the lung injury and skeletal muscle atrophy in mice Kunming mice were randomized and exposed to normal air (control) or CS at 8 h per day, 5 days per week for 12 or 24 weeks. At the end of experiment, their body, gastrocnemius muscle tissues were weighed. Their lung and gastrocnemius muscle tissue sections were stained with H&E for evaluation of the alveolar airspaces and cross-sectional areas. Data are representative images (magnification x 200) or expressed as the mean ± SD of each group (n=12 per group) of mice. **(A)** The body weights. **(B)** The gastrocnemius muscle weights. **(C)** Representative lung tissue sections. **(D)** The mean linear intercept (Lm). **(E)** Representative gastrocnemius muscle sections. **(F)** The cross-sectional area of the skeletal muscle myofibers.*p<0.05 vs. the mice after 12-week air exposure. #p<0.05 vs. the mice after 24-week air exposure.

To examine the effect of CS on the strength of skeletal muscles, male Kunming mice were randomized and exposed to CS at 5 days per week for 12 or 24 weeks. One group of mice were exposed to air and served as the control. At the end of experiment, their body and gastrocnemius weights in individual mice were measured. Their lung tissue sections were stained with H&E and examined for alveolar airspaces. The body and gastrocnemius weights in the CS-exposure mice were significantly less than that in the control mice (Figure 1A and 1B, p<0.05). The airspaces and the mean linear intercept (Lm) in the lungs of the CS-exposed mice were significantly larger than the control animals (Figure 1B and 1C, p<0.05). These indicated that long-term CS induced emphysema in mice.

Analysis of the gastrocnemius muscular sections indicated that the muscle fiber bundle of skeletal muscle cells was ranked neatly in the control mice. The skeletal muscle fibers displayed regular morphology with the myocyte nucleus in the edge of each muscle fiber (Figure 1E). In contrast, the skeletal muscle cells were arranged sparsely and loosely in the CS-exposed mice, particularly in the mice with CS exposure for 24 weeks. The cross-sectional areas of gastrocnemius muscle cells from the mice with CS exposure for 24 weeks were significantly smaller than that in the control mice (Figure 1F, p<0.05). Hence, long-term CS induced the lung injury and skeletal muscle atrophy in mice.

### Long-term CS reduces the relative levels of MHC-IIβ, HDAC2, but enhances inflammation in skeletal muscle

**Figure 2:**
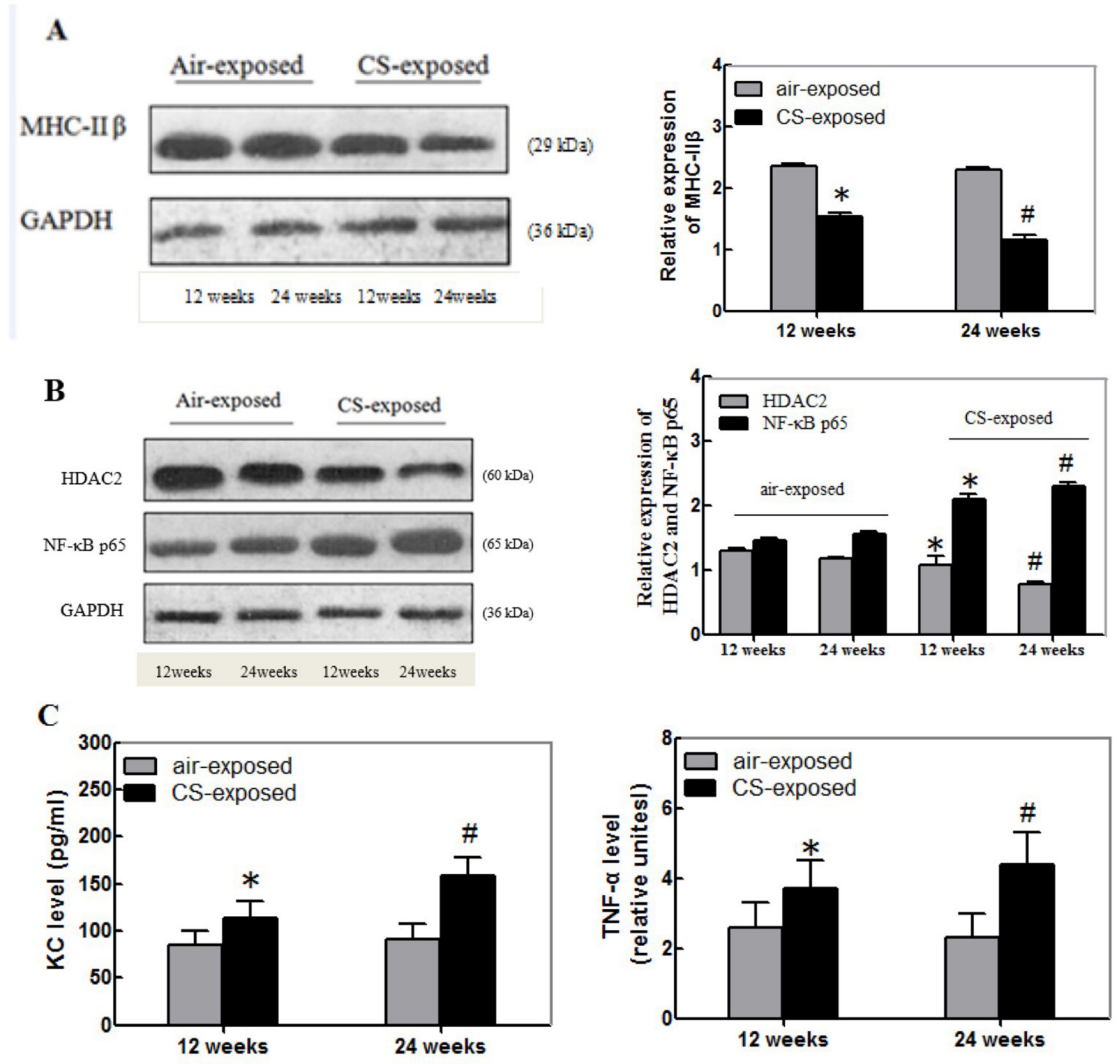
Long-term CS reduces the relative levels of MHC-IIβand HDAC2, but enhances NF-κB, KC and TNF-α expression in the gastrocnemius muscles of mice The relative levels of MHC-IIβ, HDAC2 and NF-κBp65 to the control GAPDH expression and the levels of KC and TNF-α in the gastrocnemius muscle tissue samples of individual groups of mice at 12 or 24 weeks post CS exposure were determined by Western blot and ELISA, respectively. Data are representative images or expressed as the mean ± SD of each group (n=3 for Western blot, n=12 for ELISA) of mice from three separate experiments. **(A)** Western blot analysis of the relative levels of MHC-IIβ expression. **(B)** Western blot analysis of the relative levels of HDAC2 and NF-κBp65 expression. **(C)** ELISA for the levels of KC and TNF-α. *p<0.05 vs. the mice after 12-week air exposure. #p<0.05 vs. the mice after 24-week air exposure.

The MHC-IIβ is crucial for contractile of skeletal muscles. To explore the impact of long term CS on the function of the gastrocnemius muscles, the relative levels of MHC-IIβ expression in the gastrocnemius muscle samples from individual groups of mice were determined by Western blot. As shown in Figure 2A, the relative levels of MHC-IIβ expression in the gastrocnemius muscles from the CS exposure group of mice were significantly lower than that in the controls and the decreased effect by CS tended to be time-dependent.

Long-term CS is associated with the NF-κB activation and inflammation, which are negatively regulated by HDAC2. Next, the relative levels of HDAC2 and NF-κBp65 in the gastrocnemius muscle samples from the different groups of mice were determined by Western blot assays. The relative levels of HDAC2 expression in the gastrocnemius muscles from the CS exposure mice were significantly lower than that in the control. In contrast, the relative levels of NF-κBp65 expression in the gastrocnemius muscles from the CS exposure mice were significantly higher than that in the control mice (p<0.05, Figure 2B). Further analysis indicated that the levels of KC and TNF-α in the gastrocnemius muscles from the CS exposure group of mice were significantly higher than that in the controls (p<0.05, Figure 2C). Similarly, the effects of long term CS exposure on the levels of HDAC2, NF-κBp65, KC and TNF-α expression in the gastrocnemius muscles tended to be time-dependent. Collectively, these data indicated that long-term CS tended to reduce the relative levels of MHC-IIβ and HDAC2 expression, but enhanced NF-κBp65, KC and TNF-α expression in the gastrocnemius muscles of mice.

### CSE impairs the differentiation of C2C12 myoblasts

**Figure 3:**
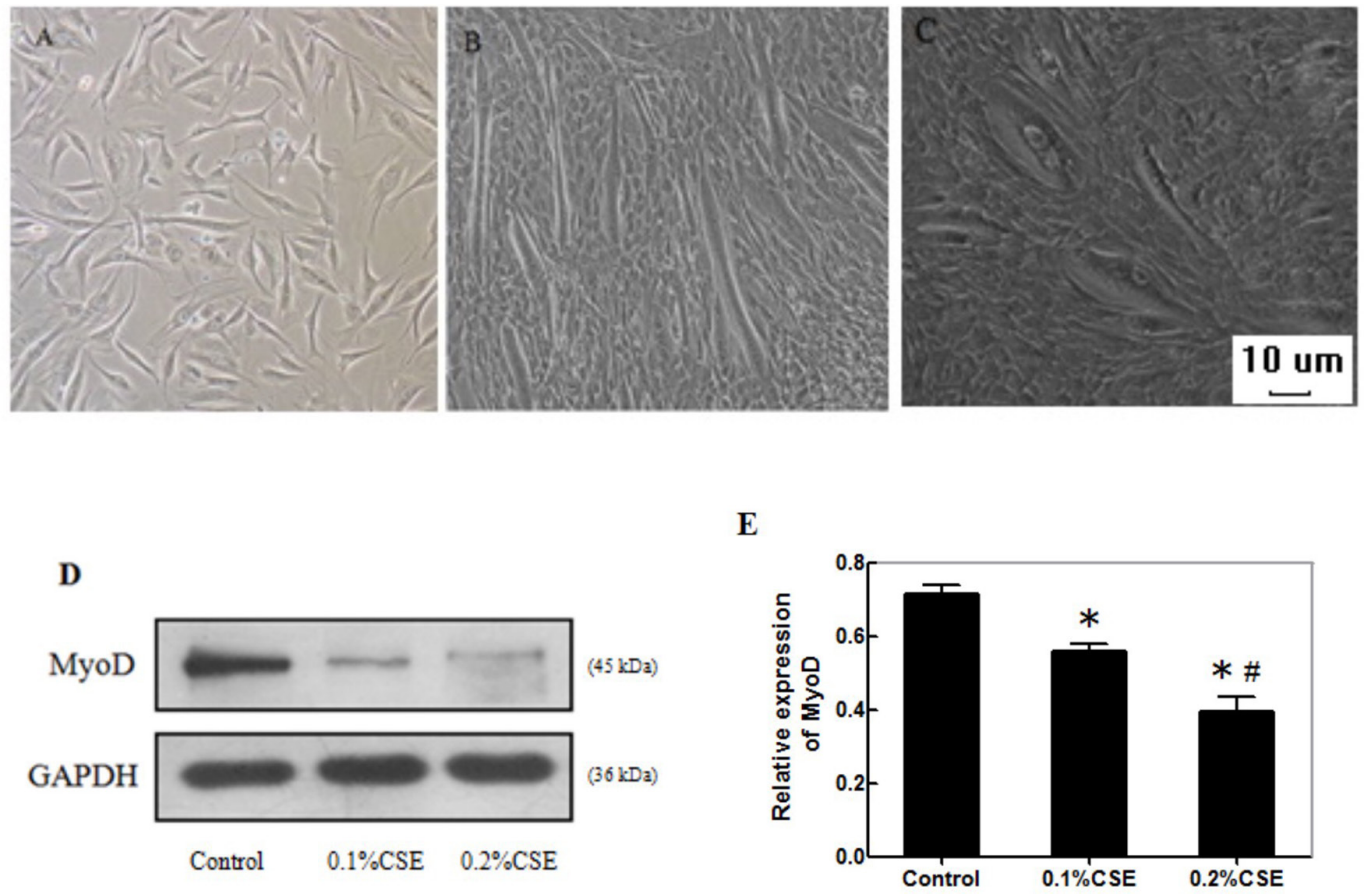
CSE reduces the myotube formation and MyoD expression during the differentiation of C2C12 cells C2C12 cells (3.0×105 cells/well) were cultured in the growth or differentiation medium for 7 days. Some cells in the differentiation condition were stimulated with 0.1% or 0.2% CSE on day 1 and 3 for 24 h. The cell morphology and myotube formation were observed under a light microscope. The relative levels of MyoD expression in the different groups of cells in the differentiation condition were determined by Western blot assays. Data are representative images or expressed as the mean ± SD of each group of cells from three separate experiments. **(A)** C2C12 cells in the growth medium. **(B)** C2C12 cells in the differentiation medium. **(C)** C2C12 cells in the differentiation medium with 0.2% CSE (magnification x 100). **(D)** Western blot analysis of the relative levels of MyoD expression. **(E)** Quantification of MyoD expression.*p<0.05 vs. the control cells, #p<0.05 vs. the cells stimulated with 0.1%CSE.

C2C12 cells can differentiate to form myotubes in a differentiation condition. To understand the effect of CS exposure on muscle atrophy, C2C12 cells were cultured in the growth, differentiation medium alone or differentiation medium containing 0.1% or 0.2% CSE. The cell morphology and myotube formation were examined. C2C12 cells in growth medium displayed single layer of a star-shape or fusiform with one central nucleus (Figure 3A). When the C2C12 cells at the differentiation condition without CSE stimulation, the cells became elongated and confluent with obvious myotubes (Figure 3B). When the cells were incubated in differentiation medium with 0.2% CSE, the cells formed sparse and short myotubes (Figure 3C).

MyoD, a myogenic regulatory factor, can activate the transcription program of muscle-specific genes to regulate the muscle differentiation. We examined the effect of CSE on the expression of MyoD in C2C12 cells in the differentiation condition by Western blot assay. We found that the relative levels of MyoD expression in the cells incubated with different concentrations of CSE were significantly lower than that in the control cells without CSE stimulation (p<0.05, Figure 3D and 3E). The inhibitory effects of CSE tended to be dose-dependent. Thus, CSE inhibited the formation of myotubes, associated with inhibition of MyoD expression in C2C12 cells.

### CSE reduces the HDAC2 expression and enhances inflammatory cytokine production in differentiating C2C12 cells

**Figure 4:**
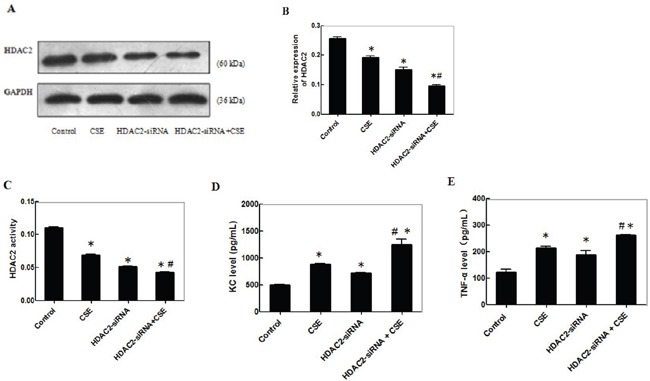
CSE reduces theHDAC2 expression and enhances inflammatory cytokine production in differentiating C2C12 cells C2C12 cells were transfected with control or HDAC2-specific siRNA for 24 h and the cells at 3.0×105 cells/well were stimulated in triplicate with, or without, 0.2% CSE for another 24 h. The relative levels of HDAC2 expression in different groups of cells were determined by Western blot and the levels of HDAC2 enzymatic activity was determined by an enzymatic assay. The levels of KC and TNF-α in the culture supernatants of different groups of cells were determined by ELISA. Data are representative images or expressed as the mean ± SD of each group of cells from 3-5 separate experiments. **(A)** Western blot analysis of the relative levels of HDAC2 expression from three separate experiments. **(B)** Quantification of HDAC2 expression. **(C)** The levels of HDAC2 activities from 5 separate experiments. **(D-E)** The levels of KC and TNF-α in the supernatants of cultured cells from 5 separate experiments.*p<0.05 vs. the control; #p<0.05 vs. the cells stimulated with CSE or with HDAC2 knockdown alone.

CSE can stimulate inflammatory cytokine production, which is negatively regulated by HDAC2. To examine the effect of CSE on inflammatory cytokine production and potential mechanisms, C2C12 cells were transfected with control or HDAC2-specific siRNA for 24 h and stimulated in triplicate with, or without, 0.2% CSE for another 24 h. The relative levels of HDAC2 expression in different groups of cells were determined by Western blot (Figure 4A). The relative levels of HDAC2 in the cells stimulated with CSE were similar to that in the cells transfected with HDAC2-specific siRNA, but were significantly reduced by 25% and 35%, respectively, compared with that in the cells transfected with control siRNA (p<0.05, Figure 4B). The relative levels of HDAC2 expression in the cells transfected with HDAC2-specific siRNA and stimulated with CSE were further significantly reduced (p<0.05). A similar pattern of HDAC2 enzymatic activity was detected in the different groups of cells (Figure 4C). Further analysis indicated similar patterns of the levels of KC and TNF-α in the culture supernatants of different groups of cells (Figure 4D and 4E). Clearly, CSE stimulation inhibited HDAC2 expression and stimulated pro-inflammatory KC and TNF-α expression in differentiating C2C12 cells.

### CSE enhances the NF-κBp65 activation and inflammatory cytokine production in differentiating C2C12 cells

**Figure 5:**
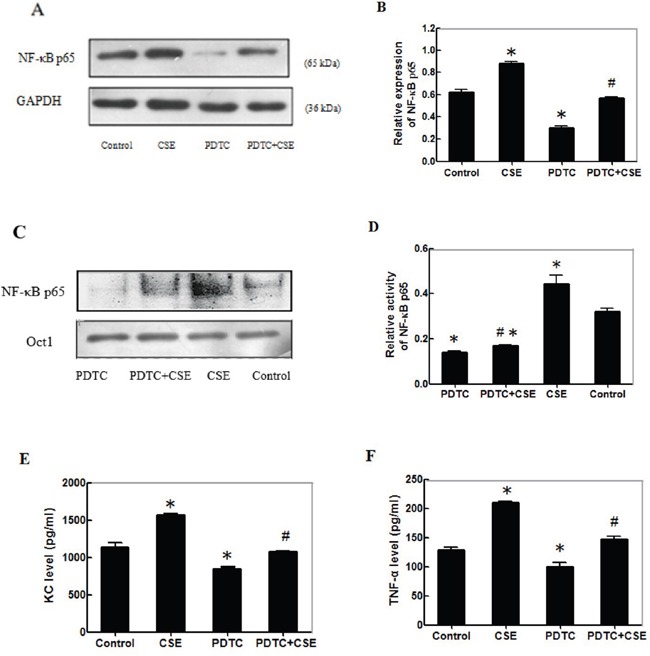
CSE enhances the NF-κBp65 activation and inflammatory cytokine production in differentiating C2C12 cells C2C12 cells (3.0×105 cells/well) were pre-treated with, or without, PDTC for 24 h and stimulated in triplicate with, or without, 0.2% CSE for another 24 h. The relative levels of NF-κBp65 expression in different groups of cells were determined by Western blot and the levels of NF-κBp65 activity was determined by EMSA using Oct 1 as the control. The levels of KC and TNF-α in the culture supernatants of different groups of cells were determined by ELISA. Data are representative images or expressed as the mean ± SD of each group of cells from 3-5 separate experiments. **(A)** Western blot analysis of the relative levels of NF-κBp65 expression. **(B)** Quantification of NF-κBp65 expression. **(C)** The levels of NF-κBp65 activity measured by EMSA. The Oct1 activity was used as the control. **(D)** Quantification of NF-κBp65 activity. **(E-F)** The levels of KC and TNF-α in the supernatants of cultured cells. *p<0.05 vs. the control. #p<0.05 vs. the cells treated with PDTC or stimulated with CSE alone.

To further understand the mechanisms underlying the action of CSE, C2C12 cells were pre-treated with the NF-κB inhibitor of PDTC for 24 h and stimulated in triplicate with, or without, CSE for another 24 h. The relative levels of NF-κBp65 expression in the different groups of cells were determined by Western blot assay (Figure 5A). The relative levels of NF-κBp65 expression in the cells treated with PDTC alone were significantly lower than that in the control (p<0.05, Figure 5B). The relative levels of NF-κBp65 expression in the cells treated with CSE were significantly higher than that in the control (p<0.05), but significantly reduced in the PTDC-treated cells. A similar pattern of NF-κBp65 activity in the different groups of cells was detected by EMSA (Figure 5C and 5D). Further analysis indicated similar patterns of the levels of KC and TNF-α in the culture supernatants of different groups of cells (Figure 5E and 5F). Together, these data indicated that CSE enhanced the NF-κBp65 activation and inflammatory cytokine production in differentiating C2C12 cells.

### CSE inhibits the interaction of HDAC2 with NF-κBp65 and promotes the accumulation of acetyl-NF-κBp65 in differentiating C2C12 cells

**Figure 6:**
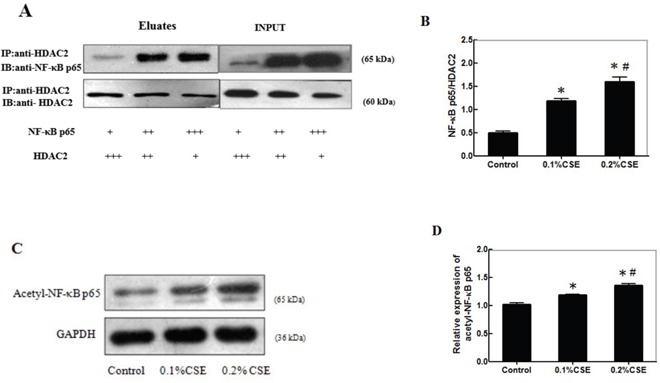
CSE inhibits HDAC2 expression and interaction with NF-κBp65 to accumulate acetyl-NF-κBp65 in differentiating C2C12 cells C2C12 cells (3.0×105 cells/well) were stimulated with, or without, 0.1% or 0.2% CSE for 24 h and the potential HDAC2/NF-κBp65complex in different groups of cells was immunoprecipitated by anti-HDAC2 antibodies. Subsequently, the contents of HDAC2 and NF-κBp65 in the immunocomplex from the different groups of cells were determined by Western blot using anti-HDAC2 and anti-NF-κBp65, respectively. The relative levels of acetyl-NF-κBp65 in the different groups of cells were determined by Western blot. Data are representative images or expressed as the mean ± SD of each group of cells from three separate experiments. **(A)** Immunoprecipitation of HDAC2 and Western blot analysis of the relative levels of NF-κBp65and HDAC2. **(B)** The ratios of NF-κBp65 to HDAC2 in the immunocomplex. **(C)** Western blot analysis of the relative levels of acetyl-NF-κBp65. **(D)** Quantification of acetyl-NF-κBp65.*p<0.05 vs. the control. #p<0.05 vs. the cells stimulated with 0.1%CSE.

Finally, we examined the effect of CSE on the interaction of HDAC2 with NF-κBp65 in differentiating C2C12 cells. C2C12 cells were stimulated with, or without, 0.1% or 0.2% CSE for 24 h and the potential HDAC2/NF-κBp65 complex in different groups of cells was immunoprecipitated by anti-HDAC2 antibodies. Subsequently, the contents of HDAC2 and NF-κBp65 in the immunocomplex from the different groups of cells were determined by Western blot using anti-HDAC2 and anti-NF-κBp65, respectively (Figure 6A). Quantitative analysis indicated that the ratios of NF-κBp65 to HDAC2 in the cells stimulated with CSE significantly higher than that in the control and the enhanced effects of CSE tended to be dose-dependent (Figure 6B). Further analysis indicated that the relative levels of acetyl-NF-κBp65 in the cells stimulated with CSE were significantly higher than that in the control and the stimulatory effects of CSE on the levels of acetyl-NF-κBp65 tended to be dose-dependent in differentiating C2C12 cells (Figure 6C and 6D). Therefore, CSE inhibited HDAC2 expression and interaction with NF-κBp65, leading to accumulation of acetyl-NF-κBp65 in differentiating C2C12 cells.

## DISCUSSION

In this study, we employed a mouse model of CS-induced pulmonary injury to explore the effect of long-term CS on the gastrocnemius muscle. Besides pulmonary injury, we found that 12-24 weeks CS-exposure could reduced the gastrocnemius muscle mass and body weights in mice. Furthermore, CS reduced the relative levels of MHC-IIβ and HDAC2 expression, enhanced the NF-κBp65, KC and TNF-α expression in the gastrocnemius muscles of mice. These novel findings provided new insights in the pathogenesis of CS-related skeletal muscle atrophy in mice.

A previous study has shown that severe COPD patients usually display fast-twitch fiber atrophy and significantly reduced levels of MHC expression in skeletal muscles [11]. In this study, we found that 24 weeks CS-exposure reduced the gastrocnemius muscle mass and body weights in mice. Our data were consistent with the notion that long-term CS not only causes lung parenchymal destruction and COPD, but also induces muscle atrophy [12]. Furthermore, we found significantly reduced levels of MHC-IIβ in the gastrocnemius muscle tissues in the CS mice. Given that the levels of MHC-IIβ are crucial for muscle function, the reduced levels of MHC-IIβ indicated an impairment of muscle function and muscle wasting in the CS mice. In addition, CSE stimulation reduced the relative levels of MyoD in differentiating C2C12 cells. It is possible that CS may inhibit the differentiation of myoblasts into myocytes *in vivo* or CS-related inflammation induces the degeneration and apoptosis of myofibers, together with many other factors, leading to muscle atrophy [13, 14].

During the progression of CS-related COPD, CS can induce systemic inflammation and stimulate pro-inflammatory cytokine, such as KC and TNF-α, production, contributing to muscle atrophy. In this study, we found that long-term CS significantly increased the levels of KC and TNF-α in the gastrocnemius muscles. Our data were in agreement with previous findings [15, 16]. Similarly, CSE stimulated KC and TNF-α secretion in differentiating C2C12 cells. Indeed, high levels of serum TNF-α are associated with loss of body weights in COPD patients [17]. However, another study indicates that lower levels of TNF-α are detected in the quadriceps muscles and similar levels of other pro-inflammatory cytokines are observed in both control and COPD patients [18]. The discrepancy may stem from varying population of patients with different severity of COPD-related muscle atrophy. More importantly, TNF-α can inhibit PGC-1α expression, leading to vascular and myocyte dysfunction [19] and IL-8 has been associated with increased resting energy expenditure and recruits inflammatory infiltrates [20]. Thus, pro-inflammatory cytokines may be important for COPD-related muscle atrophy.

The activation of NF-κB signaling is crucial for inflammation, which is negatively regulated by HDAC2 expression. We found that CS significantly increased the relative levels of NF-κBp65, but decreased HDAC2 expression in the gastrocnemius muscles of mice. Similarly, CSE stimulation increased the NF-κBp65 expression and activity, but decreased the levels of HDAC2 expression, accompanied by enhanced KC and TNF-α expression in C2C12 cells. Furthermore, knockdown of HDAC2 expression enhanced CSE-stimulated KC and TNF-α expression in C2C12 cells. Similarly, inhibition of the NF-κB signaling mitigated CSE-stimulated KC and TNF-α expression in C2C12 cells. Our findings support the notion that activation of NF-κB is important for skeletal muscle atrophy by up-regulating MuRF1 expression [21–23]. In addition, we found the direct interaction of HDAC2 with NF-κBp65 and increased the levels of acetyl-NF-κBp65 in CSE-treated C2C12 cells. These findings indicated that CSE impaired the deacetylation of NF-κBp65 by HDAC2 [24], which is associated with muscle atrophy [25]. Therefore, therapeutic enhancement of HDAC2 activity to reduce the NF-κB activation may be a new strategy for intervention of CS-related COPD and muscle atrophy.

In summary, we found that long-term CS inhibited HDAC2 expression to enhance the NF-κB activation and inflammatory KC and TNF-α production, contributing to skeletal muscle atrophy in mice. Our findings may provide new insights into the pathogenesis of COPD-related skeletal muscle atrophy.

## MATERIALS AND METHODS

### Animals

Male Kunming mice (5-6 weeks old) were purchased from the Animal Research Center of Guangxi Medical University, China and housed in a specific pathogen-free facility with a cycle of 12-h/12-h light/dark. The experimental protocol was approved by the Animal Ethics Committee of Guangxi Medical University.

### Preparation of CS extracts and CS exposure

The 0.2% CS extracts (CSE) in serum-free DMEM medium were prepared, as described previously [26]. The mice were exposed to CS as described previously [27]. Briefly, the experimental mice were exposed to the mainstream smoke in a chamber with particulate matter at 140 mg/m3 at 8 hr per day for 5 consecutive days per week for 12 or 24 consecutive weeks. The age and gender-matched control group of mice were exposed to air in the same type of chamber without smoke.

### Mouse sample collection and processing

At the end of the experiments, the experimental and control mice were sacrificed. The left lungs and left gastrocnemius muscles were dissected and used for histology [28, 29]. The right lung and gastrocnemius muscle tissues were homogenized for Western blot analysis and enzyme-linked immunosorbent assay (ELISA).

### Histology

The collected tissues were fixed in 10% formalin overnight and paraffin-embedded. The tissue sections (4 μm) were stained with hematoxylin and eosin (H&E) and photoimaged under a light microscope. The mean linear intercept (Lm) of alveolar spaces and the cross-sectional area of the gastrocnemius tissue sections from different groups of mice were analyzed in a blinded manner, as described previously [30, 31].

### Cell culture

Murine skeletal muscle C2C12 cells were cultured in DMEM medium containing 10% heat-inactivated fetal bovine serum (FBS), 2 mM glutamine, 1% antibiotics, 0.5% anti-mycoplasm and 25 mM Hepes (growth medium, Invitrogen, Carlsbad, USA) at 37°C in a 5% CO_2_ humidified atmosphere [32]. When the cells reached 80% confluency, the cells were cultured in 2% horse serum DMEM (differentiation medium) to induce differentiation for 7 days.

### Transfection and treatment

C2C12 cells (3.0×10^5^ cells/well) were cultured in six-well plate in the growth or differentiation medium for 7 days. Some cells in the differentiation medium were treated with 0.1% or 0.2% CSE on day 1 and 3 for 24 h. The cell morphology and myotube formation were examined under a light microscope. C2C12 cells (3.0×10^5^ cells/well) were cultured in the differentiation medium overnight (about 70% confluency) and transfected in triplicate with 4.0 μg HDAC2-specific siRNA (Life Technologies™, Shanghai, China) for 24 h using the SuperFect transfection reagent, according to the manufacturer's instruction (Qiagen, Valencia, USA). The sequences were sense 5′-CCACAGCGAUGAGUAUAUCAAGUUU-3′ and antisense 5′-AAACUUGAUAUACUCA UCGCUGUGG-3′ The cells were treated with, or without, 0.1% or 0.2% of CSE on day 1 and 3 for 24 h and their supernatants were harvested for ELISA analysis of KC and TNF-α. The cells were collected for immunoprecipitation and Western blot assays.

In addition, C2C12 cells were pre-treated in triplicate with, or without, 20 μM pyrrolidine dithiocarbamate (*PDTC*, the selective NF-kB inhibitor, Sigma) for 24 h and the cells were treated with CSE for another 24 h. The cells were further cultured in the differentiation DMEM for another 5 days. Their supernatants and cells were collected for ELISA, immunoprecipitation and Western blot assays.

### Enzyme-linked immunosorbent assay

The levels of KC and TNF-α in the supernatants of cultured cells were measured in triplicate by ELISA using specific kits, following the manufacture's protocols (CUSABIO, Wuhan, China). The detection limitation for KC and TNF-α is 1.25 pg/ml and 15.6 pg/ml, respectively.

### Immunoprecipitation and western blot

The collected different groups of C2C12 cells were homogenized in cell lysis buffer and the homogenized samples (30 mg/sample) were incubated with 15 μg anti-HDAC2 (Cell Signaling Technology, Boston, USA) at 4°C for 4 h. Subsequently, the mixture samples were incubated with protein A/G beads overnight at 4°C with gently rocking. After being washed, the bound proteins were eluted for Western blot assay. Briefly, the eluted proteins (30 μg/lane) were separated by sodium dodecyl sulfate polyacrylamide gel electrophoresis (SDS-PAGE) and transferred to polyvinylidene fluoride (PVDF) membranes. After being blocked with 5% fat-free dry milk in TBST, the membranes were incubated with anti-HDAC2 (1:1000 dilution), and anti-NF-κBp65 (1:1000 dilution, Cell Signaling Technology), respectively. The bound antibodies were detected with Horseradish Peroxidase (HRP)-conjugated second antibodies (ZSGB-BIO, Beijing, China) and visualized using the enhanced chemiluminescence reagent (Pierce, Waltham, USA). The relative levels of NF-κBp65 to the control HDAC2 in individual groups of cells were determined by densitometric scanning using Image J software.

In addition, the collected muscular tissues and cells were lyzed in lysis buffer. The tissue and cell lysates (30 μg/lane) were subjected to Western blot analysis using control anti-GAPDH (1:10000 dilution), anti-myosin heavy chain (MHC)-IIβ (1:1000 dilution,), anti-HDAC2, anti-NF-κBp65, and anti-acetyl-NF-κBp65 (1:1000 dilution, Cell Signaling Technology), respectively.

### HDAC2 activity assay

The HDAC2 enzymatic activity in the different groups of samples was measured in triplicate by FLUOR DE LYS®-Green HDAC2 assay using a fluorescent assay kit, following the manufacturer's instructions (Biomol, Farmingdale, USA). The principle of this assay is based on detection of fluorescent developer at a sensitivity of 1-10 ng/well. The homogenized tissue and cell samples were incubated with the HDAC substrate provided to deacetylate the substrate. The deacetylated substrate by individual samples was cleaved by the Developer to produce a fluorophore, which was easily analyzed using a fluorescence plate reader (Ex/Em=380/500 nm).

### Electrophoretic mobility shift assay

The levels of NF-κBp65 activation in the different groups of C2C12 cells were determined by electrophoretic mobility shift assay (EMSA) as described previously [33], with the 3′-end DIG-labeled oligonucleotide probe of 5′-AGTTGAGGGGACTTTCCCAGGC- 3′. Briefly, the nuclear extracts from individual groups of cells were prepared using a specific kit (Fisher), according to the manufacturers’ instruction. The nuclear proteins (10 μg/sample) were interacted in duplicate with the DNA probe in binding buffer containing poly(dI-dC) at room temperature for 15 minutes. The samples were analyzed by non-denaturing polyacrylamide gel electrophoresis and transferred onto Hybond-N+ nylon membranes (Roche). The DNA/protein complex were detected using anti-DIG and visualized using the enhanced chemiluminescence reagent. The relative levels of NF-κBp65 in the nuclear extracts of individual samples were determined.

### Statistical analysis

Data are expressed as the mean ± SD. The data distribution was analyzed by Pearson normality test. The difference among groups was analyzed by one way ANOVA and post hoc Tukey-Kramer test. The independent-samples were analyzed by Student T test. All statistical analyses were performed using the SPSS 16.0 software (SPSS, Chicago, USA). A *P*-value of < 0.05 was considered statistically significant.
